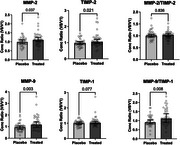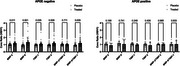# Assessing the effect of eicosapentaenoic acid (EPA) on CSF levels of matrix metalloproteinases and their inhibitors

**DOI:** 10.1002/alz70856_106642

**Published:** 2026-01-08

**Authors:** Samantha L. Shapiro, Carol A. Van Hulle, Aimee K Johnson, Hannah Zylstra, Kate Cronin, Aleshia Cole, Elena Beckman, Allison C Eierman, Richard J. Chappell, Sanjay Asthana, Carey E. Gleason, Henrik Zetterberg, Sterling C. Johnson, Anthony P Auger, Cynthia M. Carlsson

**Affiliations:** ^1^ University of Wisconsin ‐ Madison, Madison, WI, USA; ^2^ University of Wisconsin School of Medicine and Public Health, Madison, WI, USA; ^3^ Wisconsin Alzheimer's Disease Research Center, University of Wisconsin School of Medicine and Public Health, Madison, WI, USA; ^4^ Division of Geriatrics and Gerontology, Department of Medicine, University of Wisconsin School of Medicine and Public Health, Madison, WI, USA; ^5^ Geriatric Research, Education and Clinical Center (GRECC), William S. Middleton Memorial Veterans Hospital, Madison, WI, USA; ^6^ UK Dementia Research Institute, UCL Institute of Neurology, University College London, London, England, United Kingdom; ^7^ Hong Kong Center for Neurodegenerative Diseases, Hong Kong, Hong Kong, China; ^8^ Department of Psychiatry and Neurochemistry, Institute of Neuroscience and Physiology, The Sahlgrenska Academy, University of Gothenburg, Mölndal, Sweden; ^9^ Wisconsin Alzheimer's Disease Research Center, University of Wisconsin‐Madison, School of Medicine and Public Health, Madison, WI, USA; ^10^ Wisconsin Alzheimer's Institute, University of Wisconsin School of Medicine and Public Health, Madison, WI, USA; ^11^ University of Wisconsin ‐ Madison, Department of Psychiatry, Madison, WI, USA; ^12^ University of Wisconsin ‐ Madison, Neuroscience Training Program, Madison, WI, USA

## Abstract

**Background:**

Matrix metalloproteinases (MMPs) are a group of enzymes with roles in mediating extracellular matrix integrity and inflammation, and degrading beta amyloid fibrils. Studies have demonstrated that MMPs in atherosclerotic plaques can be downregulated by omega‐3 fatty acid eicosapentaenoic acid (EPA), but there has been little research looking at the effect of EPA on MMP levels in CSF. In this study, we evaluated the effect of an FDA approved high dose EPA supplement on CSF levels of MMP‐2 and MMP‐9, and MMP inhibitors TIMP‐1 and TIMP‐2.

**Method:**

Cognitively healthy VA‐eligible veterans (ages 50‐76) were enrolled in the Brain Amyloid and Vascular Effects of Eicosapentaenoic Acid Study (BRAVE, NCT02719327), a randomized, placebo‐controlled, double‐blind, parallel‐group clinical trial with subject *N* = 128, randomized in a 1:1 ratio to receive either 4g icosapent ethyl (Vascepa® IPE) or a placebo (mineral oil) daily for 18 months. Participants with suspected memory impairment, liver or kidney disease, or those unable to comply with study procedures were excluded. CSF biomarkers MMP‐2, MMP‐9, TIMP‐1, and TIMP‐2 were obtained at baseline, 9 months, and 18 months. In these exploratory analyses, treatment group differences in CSF biomarkers (18 month/baseline ratio) were analyzed on an intent to treat basis using GraphPad Prism®.

**Result:**

Contrary to published studies showing decreased levels and expression of MMP‐2 and ‐9 in individuals treated with EPA, our data revealed significant increases in levels of MMP‐2 (*p* = 0.04) and MMP‐9 (*p* = 0.003) in the IPE treated group compared to placebo. There was also a significant increase in TIMP‐2 in the IPE group (*p* = 0.02), which is consistent with previously published *in vitro* studies. Although not statistically significant, the effect of IPE seems to be modified by APOE4 status, with a trend towards decreased MMP‐2, MMP‐9, and TIMP‐2 in APOE4‐positive individuals.

**Conclusion:**

In this randomized clinical trial, veterans treated with high dose EPA (Vascepa® IPE) for 18 months demonstrated significant increases in CSF levels of MMP‐2, MMP‐9, and TIMP‐2 compared to individuals treated with placebo. These results contrast published studies showing decreased MMP levels in atherosclerotic plaques treated with EPA. Further research is needed to elucidate the effect of EPA on cerebral biomarkers.